# Unravelling the pathophysiology of diabetic foot ulcer: insights into a complex wound healing process

**DOI:** 10.3389/fcdhc.2026.1759605

**Published:** 2026-02-18

**Authors:** Mohannad N. AbuHaweeleh, Sara Ali, Yasmin Elsalakawi, Aisha Al-Khulaifi, Viviana Maggio, Manfredi Rizzo, Ammar Boudaka

**Affiliations:** 1 College of Medicine, QU Health, Qatar University, Doha, Qatar; 2 Department of Urology, Ambulatory Care Center, Hamad Medical Corporation, Doha, Qatar; 3 School of Medicine, PROMISE Department of Health Promotion Sciences Maternal and Infantile Care, Internal Medicine and Medicinal Specialties, University of Palermo, Palermo, Italy

**Keywords:** diabetes mellitus, foot ulcer, neuropathy, vascular insufficiency, wound healing

## Abstract

Diabetic foot ulcer (DFU) is a common and debilitating complication of diabetes mellitus, representing a significant clinical challenge. This article delves into the intricate pathophysiology underlying DFU, aiming to enhance our understanding of this complex wound healing process. We explore the interplay of multifactorial aspects, including peripheral neuropathy, vascular insufficiency, and impaired immune response, which contribute to the development and progression of DFU. Moreover, the dysregulation of key cellular and molecular mechanisms involved in inflammation, angiogenesis, extracellular matrix remodeling, and infection are examined. A comprehensive understanding of the pathophysiology of DFU including oxidative stress, neuropathy, dysregulated angiogenesis, impaired immune response, and key molecular pathways supports the development of targeted therapeutic strategies beyond current treatments to improve wound healing, reduce complications, and enhance patient quality of care.

## 1 Introduction

The emergence of diabetic foot ulcer (DFU) in individuals with Diabetes Mellitus (DM) stands as a distressing clinical enigma, presenting an intricate web of clinical challenges. DFU affects both the physical well-being of afflicted individuals and their quality of life (1, 2). This review aims to explain the pathophysiology of DFU.

DFU is a manifestation of an intricate interplay between multiple factors, each with its own role in the involvement of impaired wound healing (3). DFUs invariably arise from underlying pathophysiologic deformities that increase plantar pressure, where combined neuropathic and vascular impairments fail to compensate for repetitive microtrauma, ultimately leading to tissue breakdown and ulcer formation. Here, we delve into the multifaceted dynamics encompassing peripheral neuropathy, vascular insufficiency, and compromised immune responses involved in the pathophysiology of DFU.

Understanding the pathophysiology of DFU holds profound implications for the lives of individuals suffering from it (4). The knowledge that comes with understanding the causes of DFU at its molecular and cellular levels paves the way for novel therapeutic strategies that transcend the traditional, often insufficient approaches (5).

## 2 Diabetic neuropathy and DFU

### 2.1 General overview

Chronic hyperglycemia in DM can present with a debilitating complication known as diabetic neuropathy (DN). The DN interplay with other factors contributes to the development of DFU. There are several types of diabetic neuropathies each affecting certain regions in the body, but those involved in the development of DFU are peripheral neuropathies (6). The peripheral nerves that are affected include the motor, sensory and/or autonomic nerves (7).

Myelinated motor fibers are affected in a length-dependent pattern, giving us the classical distribution of peripheral neuropathy in DM, described as a glove-stocking distribution (3). As a result of this motor function loss, deep tendon reflexes are typically absent in the lower extremities, starting first with the absence of the ankle reflex, and progressing to the knee reflex, followed by loss of the reflexes in the upper extremities (8). Additionally, sensory loss plays an important role in the pathophysiology of DFU. The loss of type C sensory fibers makes the individual unable to sense pain (3). Whereas the loss of type A myelinated fibers leads to loss of proprioception, pressure sensation, vibratory perception, and subsequent abnormal gait. Consequently, individuals with DM will unknowingly experience repetitive traumas to their feet ranging from blisters to metatarsal bone fractures (9, 10).

The combination of loss of motor reflexes alongside sensory loss and inflammation eventually causes bone destruction, joint subluxation and possibly dislocation, followed by new bone formation resulting in an irreversible foot deformity known as “Charcot foot”, with the midfoot being the most affected region (11). Charcot neuroarthropathy, or Charcot foot, is a complex neurological and physiological process characterized by bone demineralization leading to progressive deformity and potential dislocation, and despite its clinical significance, no definitive cause or reliable method currently exists to identify patients who are more susceptible to developing the disorder. Furthermore, autonomic dysfunction, another crucial player in the pathophysiology of DFU, contributes by reduced sweating and altered thermoregulation, making the skin dry and susceptible to developing fissures predisposing the skin to infections (cellulitis) (12). As a result of all the aforementioned events, foot deformities and ulcers develop, and if left untreated, can lead to tissue necrosis and gangrene (3).

### 2.2 Oxidative stress and ROS production in diabetic neuropathy

**Figure 1:**
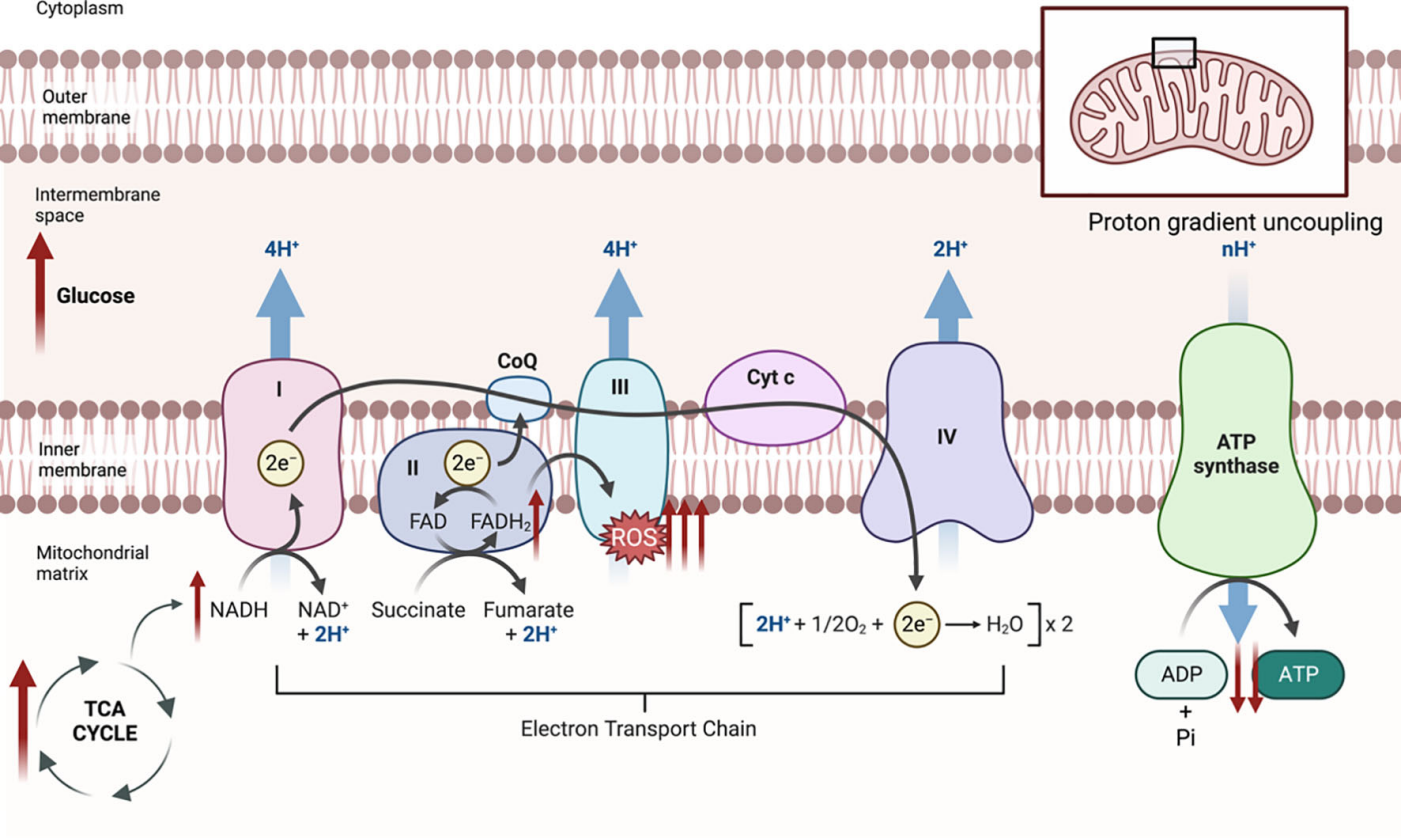
The Electron Transport Chain (ETC). In a high glucose environment, the glycolysis/TCA cycle and β-oxidation/TCA cycle increase, and an excess of NADH and FADH2 get shuttled via oxidative phosphorylation which involves the ETC. Electrons from NADH transfer from complex I to complex III, then IV, and electrons from FADH2 transfer from complex II to complex III forming ROS as a by-product, then to complex IV. As this occurs, protons move from the mitochondrial matrix into the intermembrane space, creating a proton gradient; however, due to excess glucose, proton uncoupling occurs and ATP synthase function is compromised; thus, ATP cannot be formed from ADP and Pi which hinders cellular processes.

In the primary dorsal root ganglia (DRG) and axons, glucose and fatty acids get metabolized through the glycolytic/TCA cycle and β-oxidation/TCA cycle, respectively, producing FADH2 and NADH electron donors (13). FADH2 and NADH are shuttled via oxidative phosphorylation which occurs in the inner mitochondrial membrane; this process involves the transfer and donation of electrons from NADH and FADH2 to complexes I and II, respectively (**Figure 1**) (14). The electrons then pass through complexes III and IV, simultaneously, protons are pumped from the mitochondrial matrix into the intermembrane space, creating a proton gradient (14). Protons move back into the mitochondrial matrix by the enzyme ATP synthase, and the energy created by the flow of protons drives the formation of ATP, from ADP and inorganic phosphate (15). ATP plays crucial roles in various cellular processes including the maintenance of mitochondrial function and neuronal metabolism (16). It is important to note that during electron transfer from mitochondrial complex II to complex III, small amounts of reactive oxygen species (ROS) are generated as by-products but are effectively detoxified within neurons by antioxidant systems including glutathione (GSH), catalase, and superoxide dismutase (16).

However, in chronic hyperglycemic states, the metabolism of excess glucose and fatty acids leads to a surplus of FADH2 and NADH electron donors in the DRG; thus, uncoupling the normal proton gradient (16). This hinders oxidative phosphorylation, consequently ATP production is depleted whilst ROS formation is significantly increased, and neuronal dysfunction ensues as the neuron is no longer able to counteract the ROS products via its cellular antioxidants (16, 17). Consequently, the neuron becomes overwhelmed with the excess glucose, thus the glucose or glycolysis intermediates are then shunted towards other pathways (17).

### 2.3 Molecular pathways involved in diabetic neuropathy

Several mechanisms have been postulated to play a role in the pathophysiology of DN. High levels of glucose lead to reduced insulin sensitivity, which activates the polyol pathway and promotes the formation of advanced glycation end products (AGEs). These two main pathways have been suggested to lead to the deleterious effects on mitochondrial function and increase inflammation and oxidative stress (18). Moreover, the hexosamine pathway and the protein kinase C (PKC) pathway have been shown to be involved in the development of DN (17). However, it is important to keep in mind that these pathways take part in causing multiple DM complications, such as vascular compromise and immune dysregulation, which eventually lead to DFUs.

#### 2.3.1 The polyol pathway

**Figure 2:**
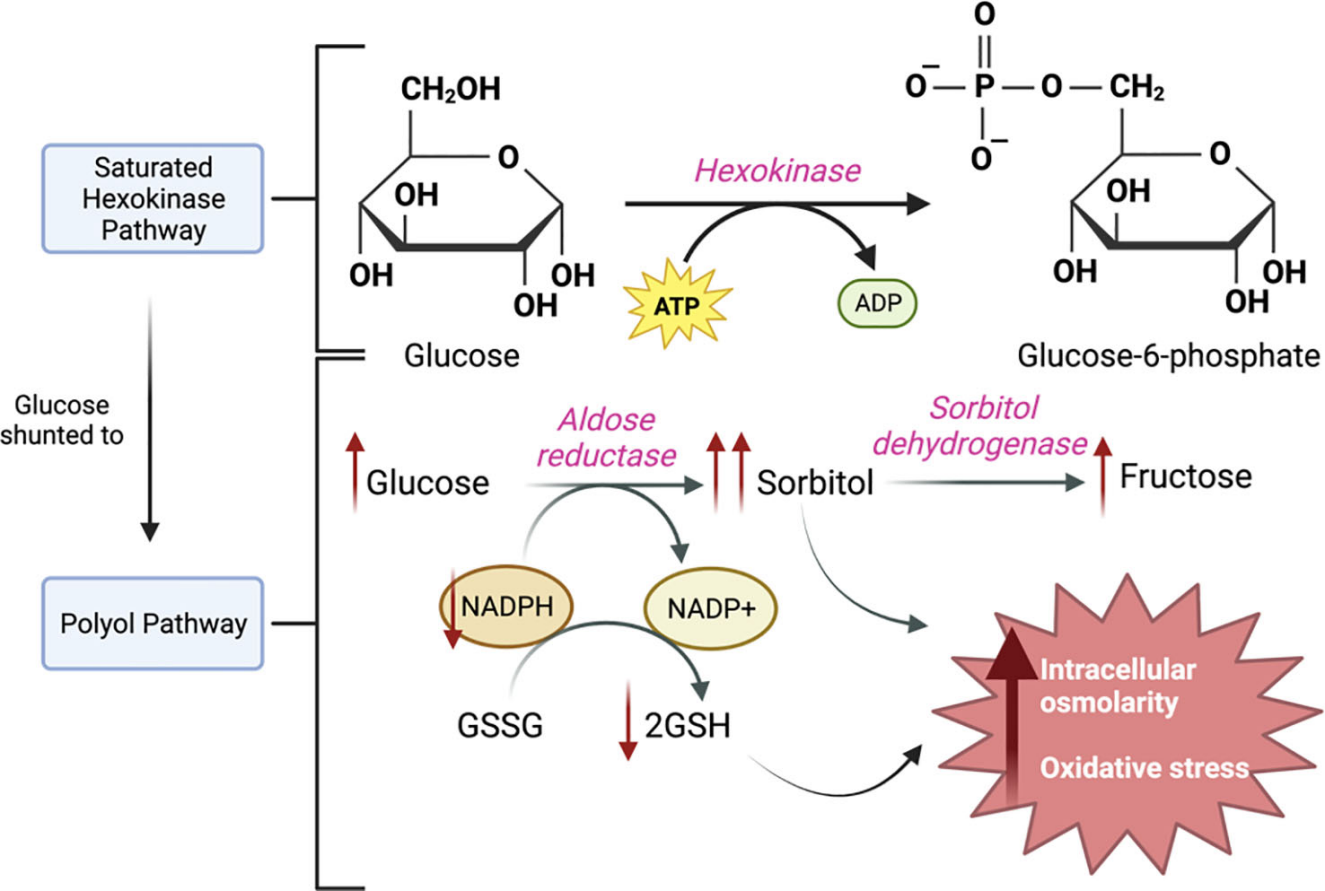
The Polyol Pathway. In a hyperglycemic state, the hexokinase pathway gets saturated, and glucose is shunted towards the polyol pathway. In the polyol pathway, glucose is converted to sorbitol by aldose reductase and NADPH is used as a cofactor, then sorbitol is converted to fructose by sorbitol dehydrogenase. As this keeps occurring, the increase in sorbitol increases intracellular osmolarity, and the decrease in NADPH and consequent decrease in GSH increases oxidative stress.

Under normal physiological conditions, glucose is converted to glucose-6-phosphate via the hexokinase pathway, but as glucose excess occurs, the hexokinase pathway becomes saturated, and glucose is shunted into the polyol pathway (19) (**Figure 2**). In this pathway, glucose is converted to sorbitol by aldose reductase and then to fructose by sorbitol dehydrogenase (19). As this reaction keeps taking place within the cell, sorbitol production surpasses its conversion to fructose, and sorbitol accumulates intracellularly (20). Due to sorbitol’s properties, it increases the intracellular osmolarity; therefore, pulls fluid into the cell, hence negatively affecting tissue osmotic homeostasis (20). Additionally, the first step in this metabolic pathway involves the oxidation of the enzyme cofactor nicotinamide adenine dinucleotide phosphate hydrogen (NADPH); thus, decreasing its availability (21). NADPH is required in a reaction involving the enzyme glutathione reductase to regenerate GSH, an essential antioxidant within the cell (21) and its depletion causes an increase in oxidative stress (22).

Schwann cells, the major glial cell type in the peripheral nervous system, have several crucial functions including the development and myelination of peripheral nerves, along with other functions. It has been observed that Schwann cells’ apoptosis was increased upon exposure to a high glucose concentration environment in both *in vitro* and *in vivo* experimental models (23).

#### 2.3.2 Advanced glycation end products pathway

**Figure 3:**
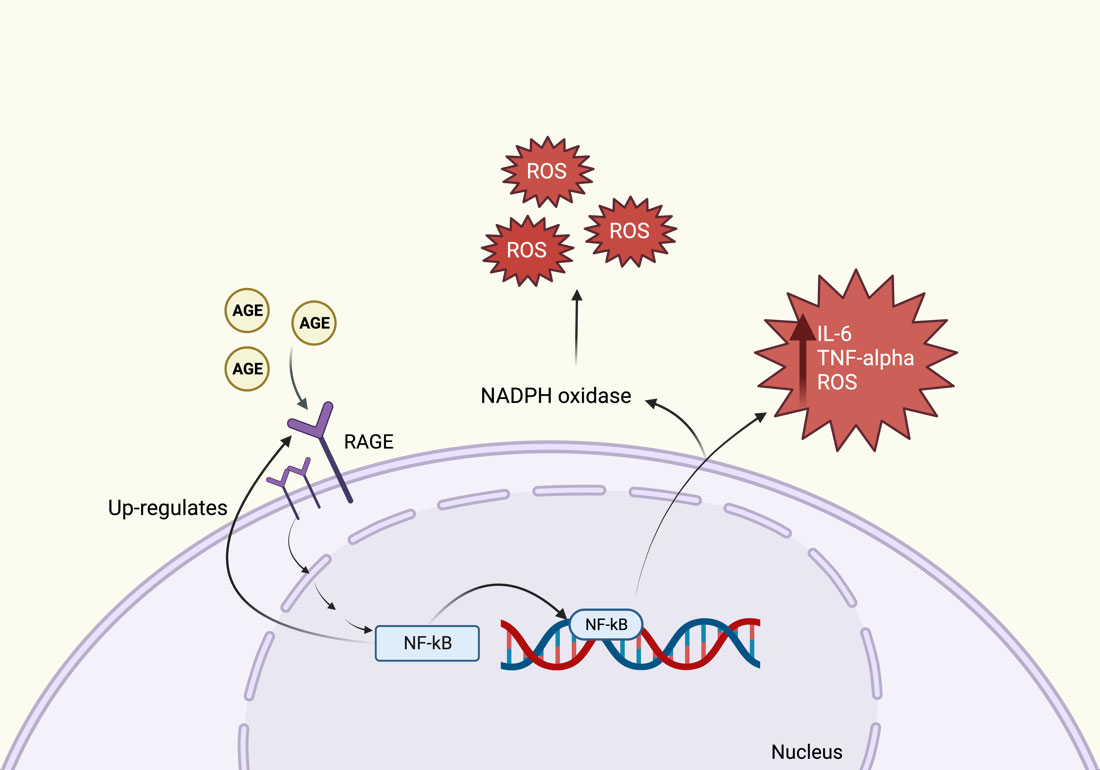
The AGEs Pathway. AGEs bind onto RAGE which triggers an intracellular pathway forming NF-kB. NF-kB causes an increase in the formation of IL-6, TNF-alpha and ROS. Formation of NADPH oxidase also contributes to an increase in ROS. NF-kB also leads to further up-regulation of RAGE.

AGEs are stable irreversible products formed by the non-enzymatic combination of a carbonyl group of reducing sugars with amine groups on lipids, proteins, and nucleic acids (24). AGEs play an important role in further increasing inflammation through binding to cell surface receptors for AGEs (RAGE) (**Figure 3**). In rodents, RAGE has been shown to be expressed in the DRG, Schwann cells and peripheral nerves (7). Once AGEs bind onto their receptors, a downstream signaling pathway is activated that is mediated partly by nuclear factor kappa B (NF-κB) (16). This triggers the release of potent pro-inflammatory cytokines such as IL-6 and TNF alpha, as well as ROS within the neurons (16). A vicious cycle occurs within the nerve, where the activation of NF-κB leads to the upregulation of RAGE, further promoting inflammation and neuronal damage (7). Alternatively, RAGE can promote the formation of ROS through activating NADPH oxidase. The elevated levels of ROS can lead to deleterious effects on DNA, proteins, and lipids, which further compromises normal neuronal function (7). Additionally, some proteins in the cytoskeleton of axons could be modified by AGEs, these include tubulin, actin, and microfilaments. Consequently, the axons lose their ability for axoplasmic transport with subsequent axonal degeneration (25). In *in vitro* experimental models, glycation of the Na^+^/K^+^ ATPase can alter the channels’ function; thus, slowing motor nerve conduction velocity (26). Moreover, macrophages release proteases to phagocytize glycated myelin, which can contribute to nerve demyelination (25).

#### 2.3.3 The hexosamine pathway

Another pathway that can be involved in the pathophysiology of DN is the hexosamine pathway. Approximately 5% of the intracellular glucose will enter the hexosamine pathway in healthy individuals (27). In this reaction, fructose-6-phophate and glutamine are converted into glucosamine-6-phosphate and glutamate by the enzyme glutamine fructose-6-phosphate aminotransferase (GFAT) (28). Glucosamine-6-phosphate can potentially increase the production of H_2_O_2_; thus, further increasing oxidative stress (29). The final step involves the metabolism of glucosamine-6-phosphate into uridine diphosphate N-acetylglucosamine (UDP-GlcNAc), this is the substrate for O-GlcNAc transferase (OGT) (28). It has been shown that gene expression, particularly of transcription factors, could be altered as result of protein modification by GlcNAc, such as the over-expression of genes involving plasminogen activator inhibitor-1 (PAI-1) and transforming growth factor-1 (TGF-1) (17, 30). PAI-1 causes smooth muscle proliferation, eventually leading to atherosclerosis, whilst TGF-1 is involved in fibrosis and inhibition of mesangial cell proliferation (30). However, the exact peripheral nerve proteins affected by this pathway remain unknown; hence, warrants further exploration (17).

#### 2.3.4 The protein kinase C pathway

An alternative reaction for fructose-6-phosphate involves its conversion to diacylglycerol (DAG), which could activate PKC. This can lead to the over-expression of NADPH oxidase; therefore, further increasing the oxidative stress through increasing the production of ROS leading to neuronal damage (29). DAG can also activate PAI-1, TGF and vascular endothelial growth factor (VEGF) (30). The over-expression of these genes is known to contribute to the devastating microvascular complications in DM, specifically diabetic retinopathy and nephropathy; however, their role in DN remains unclear (30). Currently, it is understood that the activation of the PKC pathway can alter blood vessels vasoconstriction and capillary permeability, and can cause hypoxia, new blood vessels formation, endothelial proliferation and thickening of the basement membrane; hence, it is postulated that PKC’s involvement in DN is likely due to the changes that occur to neurovascular blood flow (30). In streptozotocin (STZ)-induced diabetic rats, the inhibition of PKC was shown to prevent the development of neuronal dysfunction in rats, which could suggest an association between the PKC pathway and the development of DN (31). The interaction between all these different pathways and their mechanisms of causing neuronal injury and dysfunction, eventually lead to DN. If left unmanaged, DN, along with other factors, can lead to the development of DFU.

## 3 Vascular insufficiency and DFU

DM has a major effect on the vascular system, leading to several manifestations, including ischemia to the lower extremities. Such ischemia is believed to be one of the risk factors contributing to the progression of DFU (32, 33). It is believed that reduced blood supply to the foot will hamper ulcer healing, leading to gangrene and if the situation severely worsens, amputation may become a must (9). It is important to note that pure ischemia, with no associated neuropathy, is only responsible for almost 10% of DFU cases (34). Simultaneously, ischemia is estimated to be present in around 49-50% of DFU cases (9, 34). Ischemia, in the lower limbs, is usually the result of atherosclerotic macrovascular disease, microvascular dysfunction, and dysregulated angiogenesis (32, 33, 35). The different mechanisms leading to these events as well as their consequences will be discussed in this section.

### 3.1 Macrovascular disease

In DM, macrovascular disease manifests as peripheral vascular disease (PVD). The hallmark of PVD in DM is atherosclerosis (36) which commonly affects the tibial and peroneal arteries (3, 33). In normal blood vessels, atherosclerosis is inhibited and discouraged by the endothelial lining. This is attributed to several endothelial characteristics, including impermeability, secretion of protective substances [e.g., nitric oxide (NO)], and having a smooth thromboresistant surface (37). It is assumed that the disruption of the anatomical and functional integrity of this endothelial barrier is the main culprit in the development of atherosclerosis (36).

**Figure 4:**
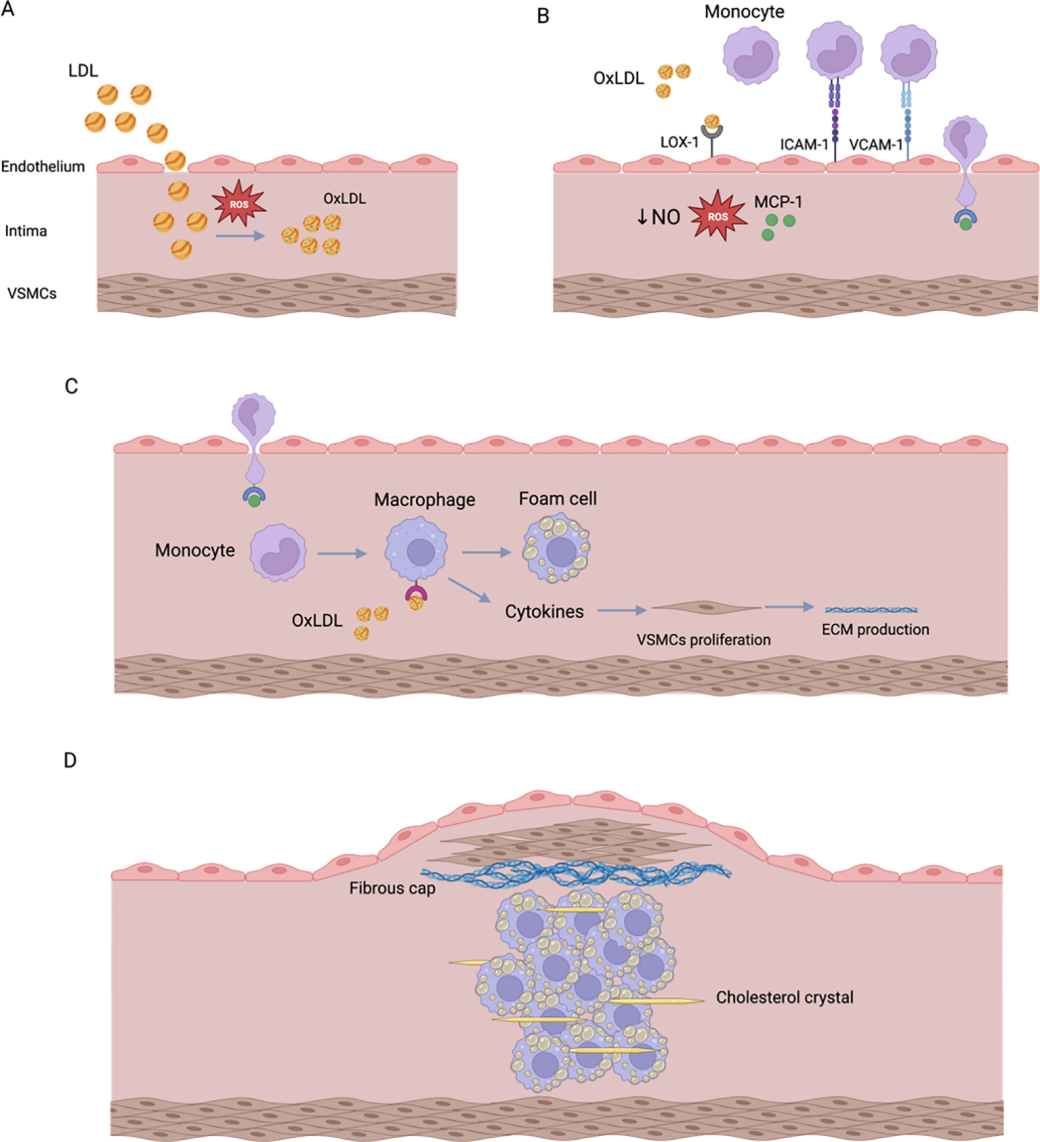
Summary of atherosclerosis development. **(A)** LDL particles enter the intima due to the increased endothelial permeability. These LDL particles will be oxidized by ROS to form OxLDL. **(B)** OxLDL will bind to LOX-1 to increase ROS production and decrease NO production. This binding will also induce the production of MCP-1, VCAM-1, and ICAM-1. These molecules will attract monocytes to the blood vessel’s intima. **(C)** Monocytes will then differentiate into macrophages, which will engulf OxLDL to form foam cells. Macrophages will also produce cytokines to induce the proliferation of VSMCs. **(D)** VSMCs will produce ECM that forms a fibrous cap to stabilize the atheroma.

The pathophysiology of atherosclerosis is almost the same in patients with and without DM. In essence, a huge amount of LDL enters the intima due to the increased endothelial permeability, allowing ROS to oxidize LDL. The oxidized LDL (OxLDL) then inhibits NO production by endothelial cells and upregulates growth factors, chemotactic proteins, and adhesion molecules. All of these will attract monocytes to the blood vessel’s intima. Once in the intima, monocytes will differentiate into macrophages which will form foam cells by interacting with OxLDL. Macrophages will also produce proinflammatory cytokines that induce the proliferation of vascular smooth muscle cells (VSMCs). In turn, VSMCs will produce ECM that forms a fibrous cap and stabilizes the atheroma (**Figure 4**). Eventually, the formed plaque can rupture and in such cases thrombosis may occur (38). It is important to note that VSMCs and the produced ECM are essential to stabilize the plaque. However, in DM, the number of VSMCs is reduced. Additionally, OxLDL, ROS, and upregulation of PKC and RAGE promote apoptosis of VSMCs. The last two may also impair the synthesis of collagen. Accordingly, DM increases the risk of plaque rupture and subsequent thrombosis (39).

**Figure 5:**
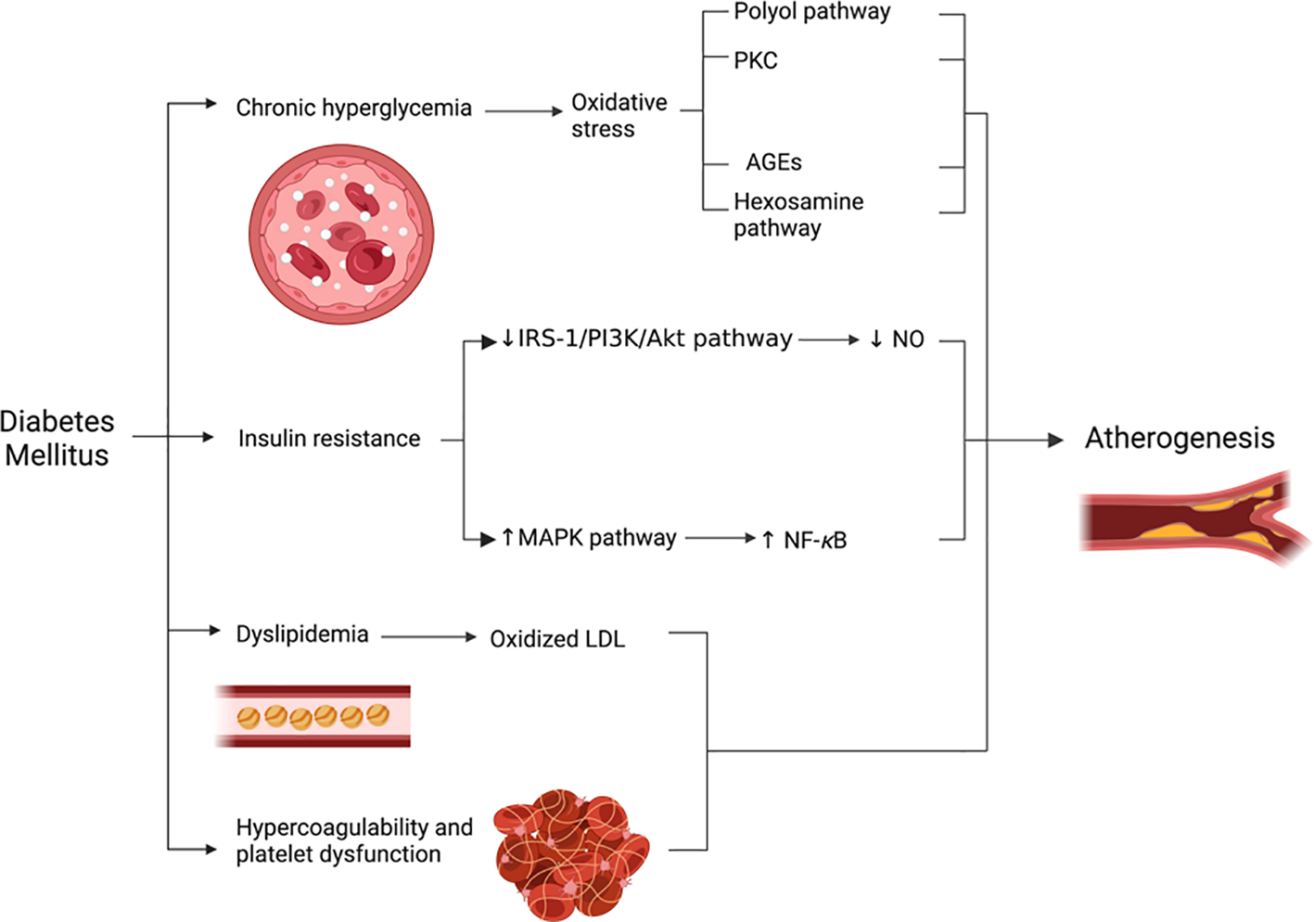
Factors contributing to the development of atherosclerosis in DM.

In the following section, the most crucial factors contributing to the development of atherosclerosis in DM will be discussed. These factors are also summarized in **Figure 5**.

#### 3.1.1 Chronic hyperglycemia and oxidative stress

As previously explained in this article, chronic hyperglycemia leads to the excessive production of ROS (40). In turn, the accumulated ROS form peroxynitrite by interacting with NO. Peroxynitrite, which is a potent oxidant, would then inactivate endothelial NO synthase (eNOS) (34). As a result, synthesis of NO decreases, while ROS production increases (32). Moreover, the four metabolic pathways involved in the pathophysiology of DN are thought to have a significant impact on the vascular system as well. This occurs via increasing endothelial permeability, reducing NO production, increasing the oxidative stress, and upregulating proinflammatory substances (e.g., NF-κB) (30, 32, 34, 35). In turn, NF-κB would stimulate the expression of intercellular adhesion molecule 1 (ICAM-1), vascular cell adhesion molecule 1 (VCAM-1), and E-selectin (35). All the aforementioned substances would promote the development of atherosclerosis by stimulating the migration of monocytes and VSMCs into blood vessels’ intima resulting in foam cell formation (41) (**Figure 4**).

#### 3.1.2 Insulin resistance

Under normal physiological conditions, insulin stimulates glucose transport and NO production via the IRS-1/PI3K/Akt pathway (42). However, in insulin resistant states, insulin cannot activate this pathway, reducing the production of NO. Concurrently, the mitogen-activated protein kinase (MAPK) pathway remains sensitive to insulin activation (42, 43). The activation of MAPK stimulates inflammatory pathways (e.g., NF-κB), as well as VSMCs growth and proliferation (42). In brief, insulin resistance decreases NO generation while increasing production of proinflammatory substances. Consequently, insulin resistance is an optimal environment for atherogenesis to develop and progress (42).

#### 3.1.3 Dyslipidemia

Patients with DM frequently have elevated levels of LDL triggering the oxidative stress-mediated lipid peroxidation, which results in OxLDL transformation. In turn, OxLDL promotes atherosclerosis via several mechanisms (44). OxLDL exerts its effects primarily by binding to lectin-like oxidized low-density lipoprotein receptor-1 (LOX-1) which is expressed in endothelial cells, smooth muscle cells and macrophages. Under normal physiological conditions, LOX-1 expression is relatively low, however, it can be induced by OxLDL, oxidative stress, and inflammatory cytokines (45).

The interaction between OxLDL and LOX-1 activates NADPH oxidase, which rapidly increases ROS generation (44). It is hypothesized that this interaction induces monocyte chemoattractant protein-1 (MCP-1), which recruits monocytes (45, 46). In addition, endothelial adhesion molecules such as E-selectin, P-selectin, VCAM-1, and ICAM-1 are produced by the same interaction (45) (**Figure 4**). OxLDL can also reduce the production of NO by displacing eNOS from the caveolae membrane and by increasing ROS production. It was proposed that LOX-1 plays a significant role in this process (46). Finally, OxLDL activates caspase-9, caspase-3, and Fas which collectively promote endothelial cell apoptosis (44).

#### 3.1.4 Hypercoagulability and platelet dysfunction

DM can induce a hypercoagulable state by increasing the levels of PAI-1 and reducing fibrinolytic activity. DM can also increase the levels of tissue factor and factor VIIA while decreasing the levels of antithrombin III and protein C (38, 39). In platelets, DM decreases NO production, increases oxidative stress, upregulates P-selectin along with glycoprotein Ib and IIb/IIIa receptors. These will collectively enhance platelet aggregation and adhesion (39). Eventually, these factors combined would destabilize the atherosclerotic plaque and subsequently increase the risk of atherothrombosis (39).

### 3.2 Microvascular dysfunction

For some time, it was believed that the predominant pathology in the microcirculation of a diabetic foot was occlusion, however, this turned out to be a misconception. The current hypothesis suggests that microcirculation is affected structurally and functionally with no significant occlusion (47).

**Figure 6:**
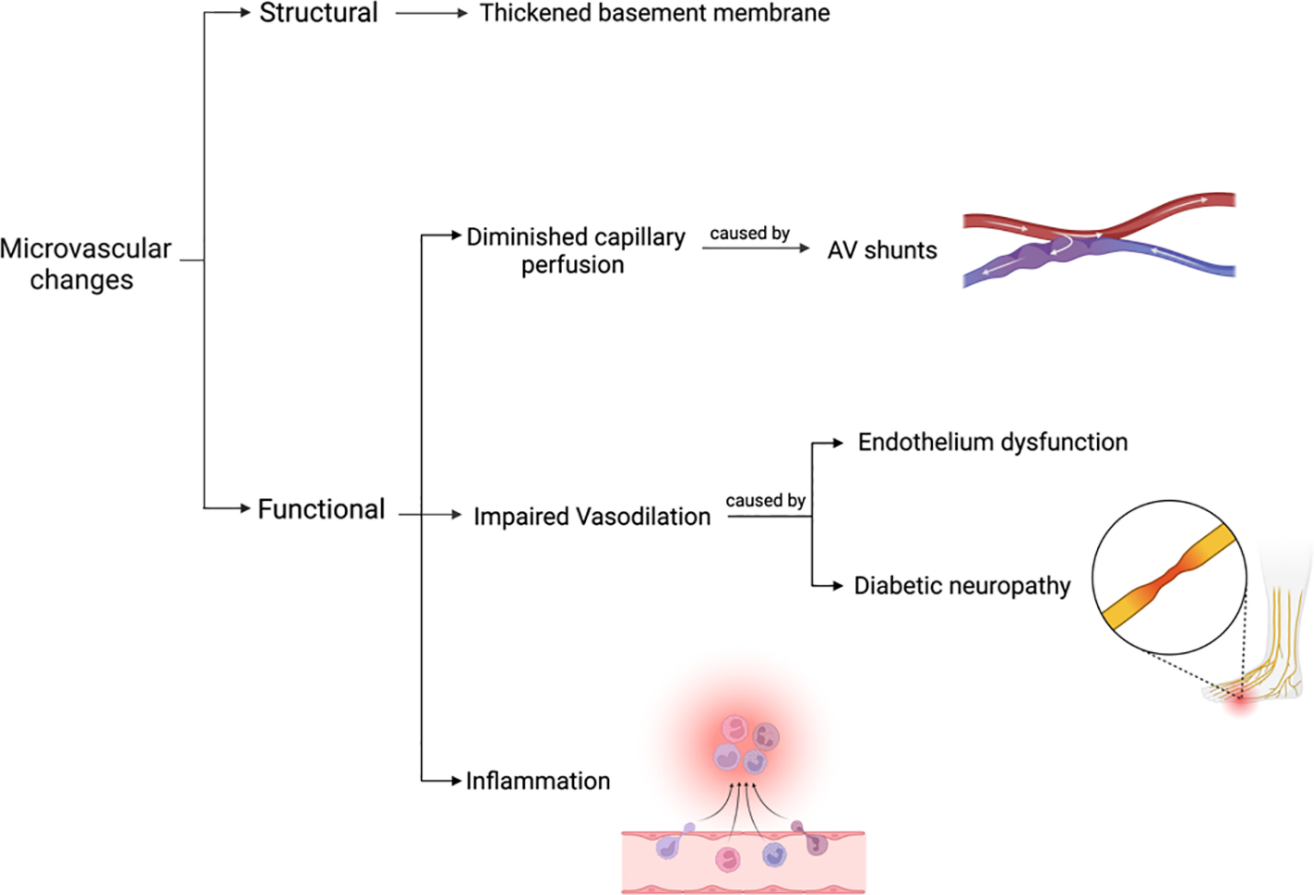
Structural and functional changes of the microvasculature in the diabetic foot.

The main structural change in capillaries is a thickened basement membrane, which is more pronounced in the lower extremities (47, 48). This may be due to the increased hydrostatic pressure which increases shear force in the capillaries. Consequently, the endothelium would increase the production of ECM proteins which thicken the basement membrane; thus, reducing leukocytes migration and hyperemic response. As a result, the diabetic foot is more susceptible to infections (47, 48). On the other hand, functional changes in the microcirculation are more extensive and include mainly three aspects as demonstrated in **Figure 6**.

#### 3.2.1 Capillary perfusion

It is assumed that the diabetic foot has blood maldistribution (48) mainly due to the presence of arteriovenous (AV) shunts (47, 48). This means that oxygen-rich arterial blood bypasses the nutritional capillaries and goes to the veins directly. This causes the foot capillaries to have a diminished perfusion (47). It is important to note that the presence of autonomic neuropathy and sympathetic denervation may augment the AV shunts and further exacerbate the maldistribution of blood (48) which can result in impaired gas exchange, nutritional delivery and removal of waste in the affected foot (47).

#### 3.2.2 Impaired vasodilation

Normal blood vessels exhibit a phenomenon called endothelium-dependent vasodilation. NO is a potent vasodilator that can induce such a phenomenon under normal conditions. As noted earlier in this chapter, endothelium dysfunction and reduced NO production are evident in DM due to various reasons. Consequently, endothelium-dependent vasodilation in the diabetic foot is impaired (47).

The nervous system is considered another key player in dilation of blood vessels and subsequent increase in the blood flow. The nervous system does this by activating C nociceptive nerve fibers which causes antidromic activation of neighboring C fibers. Then, C fibers stimulate the secretion of substance P, calcitonin gene-related peptide (CGRP), and histamine. These molecules would, in turn, promote vasodilation and increase blood flow to the tissue (48). Accordingly, an intact nervous system is necessary to promote maximal microvascular vasodilation in cases of stress (47). However, such a mechanism is diminished in patients with DN. In such a case, the foot would have reduced blood supply, especially in cases of injury, leaving the foot more susceptible to serious complications (48).

#### 3.2.3 Inflammation

As aforementioned, hyperglycemia-induced oxidative stress can activate four metabolic pathways. The overactivation of these pathways collectively induces inflammation and disrupts the integrity of the endothelial cells in capillaries. Thus, increased microcirculatory dysfunction commences (47).

### 3.3 Dysregulated angiogenesis

Hypoxia is a contributing factor that leads to poor healing of DFU, and diabetic wounds have insufficient angiogenesis. A number of studies investigated the process behind the diminished restoration of vasculature in diabetic wounds and suggested that endothelial progenitor cells (EPC) mobilization and homing are hampered, and that the amount of VEGF, the key pro-angiogenic factor in wounds, is reduced in DM (49, 50).

The formation of new blood vessels is a crucial aspect of tissue repair as this is what provides essential nutrition and oxygen to cells at the wound site. Both angiogenesis, which involves the sprouting of capillaries from existing blood vessels, and vasculogenesis, which results in the mobilization of bone marrow-derived EPC, contribute to this process (51). However, in DFU, inadequate local angiogenesis is believed to be a significant factor contributing to impaired healing. In chronic wounds of patients with DM, proteins with antiangiogenic properties, like myeloperoxidase, are expressed at higher levels compared to acute wounds, while angiogenic stimulators, such as extracellular superoxide dismutase, are generally decreased. This reduction in angiogenesis leads to increased cell death, as indicated by the expression of the late apoptotic cell marker annexin A5, which is found exclusively in diabetic wound exudates, which suggests a lack of proper wound nutritional supply (51, 52).

## 4 Impaired immune response and DFU

### 4.1 General overview

**Figure 7:**
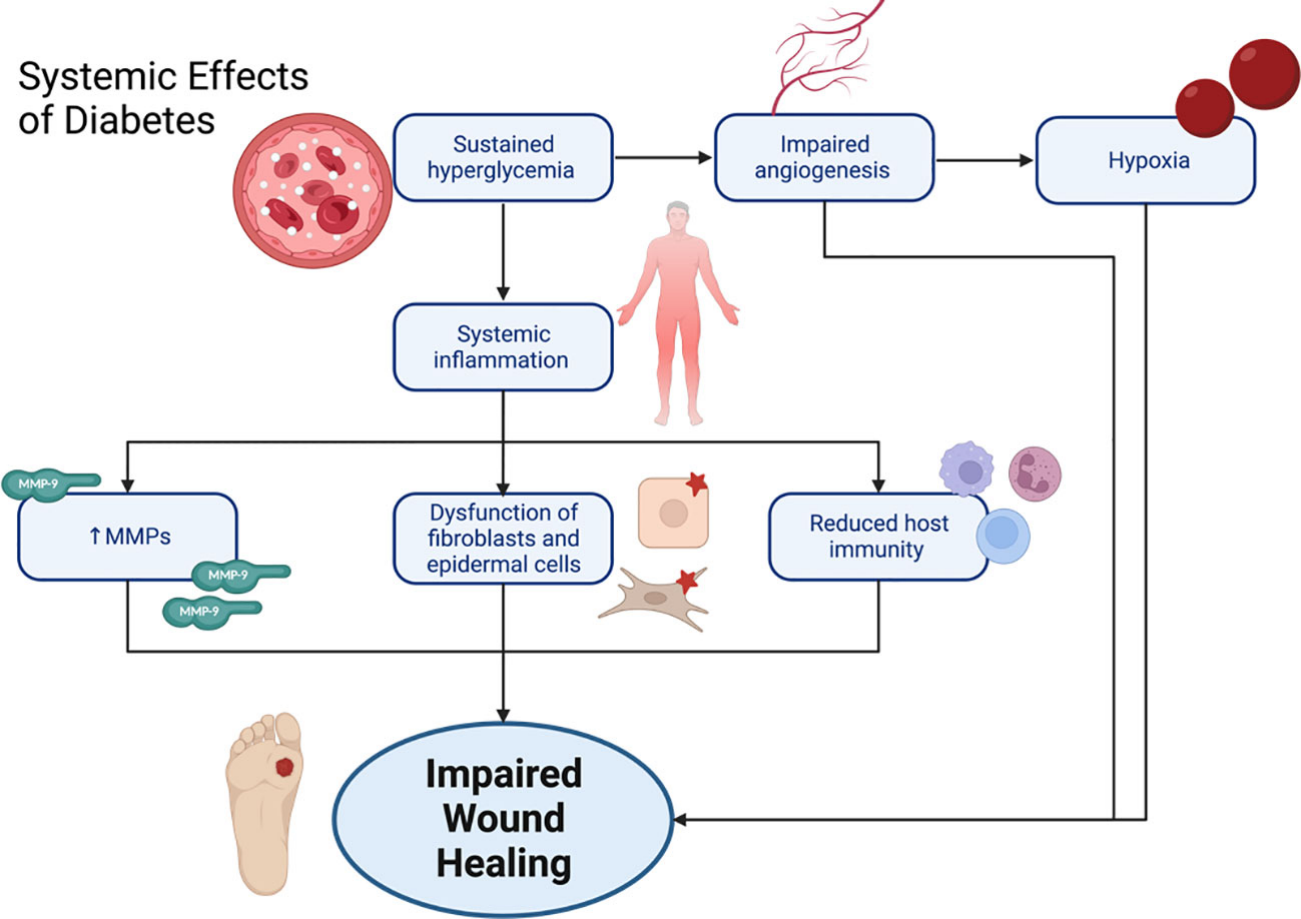
Systemic effects of diabetes leading to impaired wound healing.

Impaired host defenses and immunosuppression in DM also contribute to ulcer development and delayed wound healing. The immunological aspect of DFU involves the dysregulation of key cellular and molecular mechanisms that include inflammation, angiogenesis, ECM remodeling, and infection (**Figure 7**). These mechanisms are mediated through various immune cells and cytokines (49, 53) which normally coordinate a well-orchestrated series of events during the normal wound healing process. The key cells involved at different stages include platelets, mast cells, neutrophils, monocytes/macrophages, lymphocytes, fibroblasts, keratinocytes, epithelial cells, endothelial cells, and matrix metalloproteinases (MMPs). However, in the context of DM they become disrupted resulting in prolonged and compromised healing (50).

As discussed earlier in this article, AGE-RAGE interaction is involved significantly in the pathophysiology of DFU (50). Impaired immune response, particularly the sequelae of AGEs binding to their receptor with consequent reduction of transforming growth factor beta (TGF-β) and fibroblast growth factor (FGF), disrupts the normal mechanism of ECM formation and deposition of basement membrane components at wound site (54) maintaining prolonged inflammation (26). Moreover, this proinflammatory state is also achieved through the downstream signaling of NF-κB that yields the release of proinflammatory cytokines, including IL-1, IL-6, tumor necrosis factor alpha (TNF-α), chemokines, adhesion molecules, like VCAM-1 and the gene that codes for ICAM-1, which in turn sustains a proinflammatory environment (53, 54).

### 4.2 Stages of wound healing and DFU

**Figure 8:**
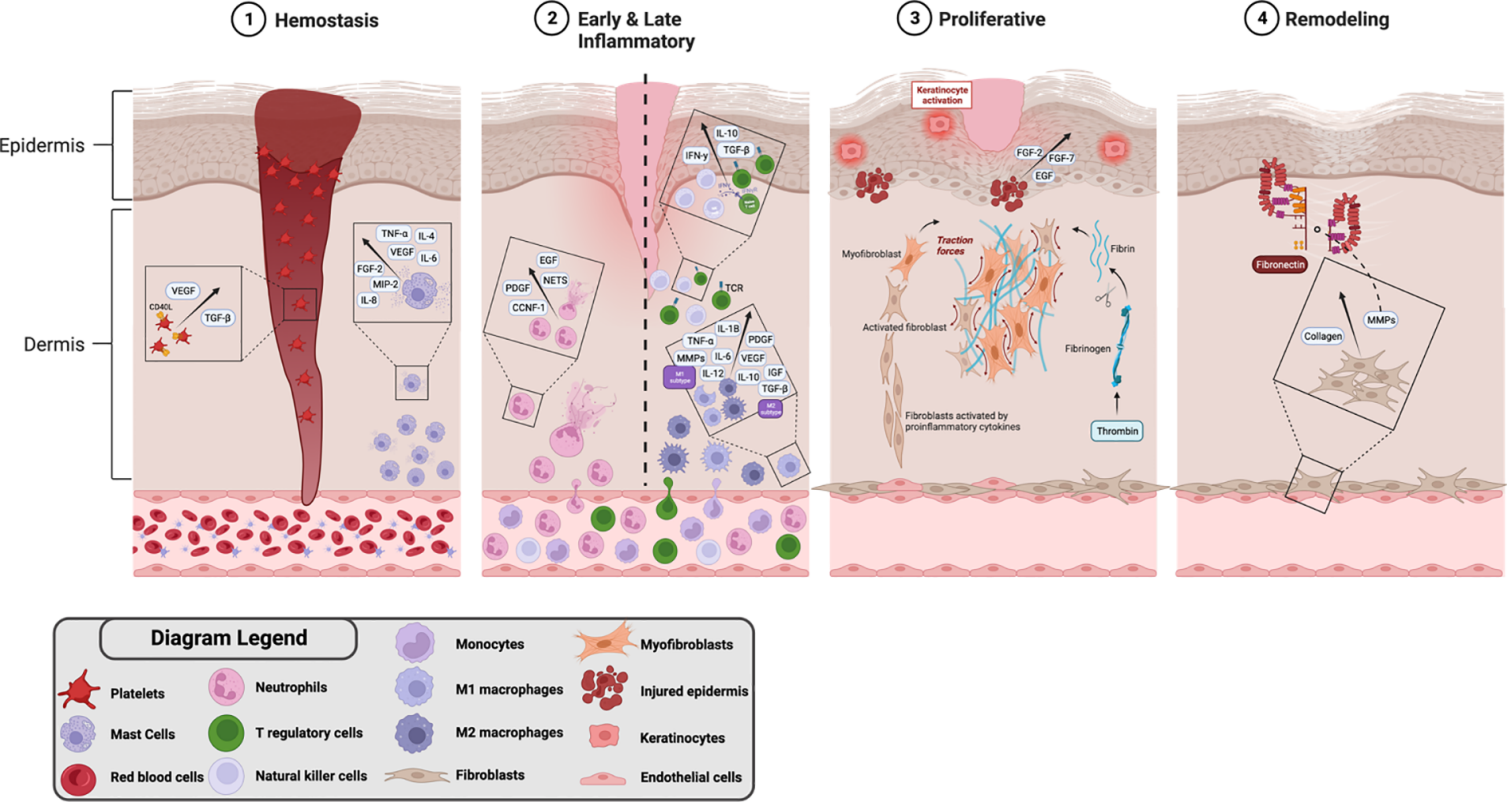
Stages of wound healing and involved key immune cells in DFU.

During the normal wound healing process there are four closely interconnected and overlapping stages: hemostasis, inflammation, proliferation, and remodeling (**Figure 8**) (50, 55–58). It is crucial for these stages to occur in a specific sequence, at appropriate times, and with optimal intensity for a designated duration. Wounds with compromised immune response tend to deviate from the regular progression of the wound healing stages. These wounds may experience pathologic inflammation, which is a result of postponed, incomplete, or poorly coordinated healing processes. This can be observed in wounds, such as ulcers, associated with chronic conditions like DM (50). In the following section, we will discuss the impact of DM on different stages of wound healing with special focus on DFU.

#### 4.2.1 Hemostasis phase: platelets and mast cells

During this phase, platelets tend to predominate within minutes after cellular injury; releasing growth factors that aid in wound healing such as platelet-derived growth factor (PDGF), TGF-β (55), and proteins, which include CD40L, CXCL4, CCL5, that initiate migration and adhesion of monocytes and neutrophils (**Figure 8**) (59). Mast cells also contribute to the wound healing process via degranulation (53, 55, 60) thereby releasing certain proinflammatory cytokines and chemokines, these include TNF-a, FGF-2, MIP-2, IL-8, IL-4, IL-6, and VEGF (56). These secretory products, especially IL-8, are involved in neutrophil recruitment (55, 56), ECM modulation via proteosome release (55, 60), and stimulation of fibroblast proliferation by the release of IL-4, VEGF, and FGF-2 (60).

It has been demonstrated in *in vitro* studies that keeping mast cells in a stabilized state contributes to normal wound healing (53, 60). In diabetic wounds there is an increase in mast cell degranulation, which is associated with the upregulation of proinflammatory cytokines, IL-6 and TNF-a (53, 60). Hence, the instability of mast cell degranulation can potentially be a contributing factor to the delayed wound healing seen in DFU.

#### 4.2.2 Early inflammation phase: neutrophils

Throughout the early phase of inflammation, neutrophils are the initial cells to arrive to injured tissue (55). Normally, Neutrophils release proinflammatory molecules including cellular communication network factor 1 (CCNF-1), PDGF, epidermal growth factor (EGF) (49). Neutrophils also release neutrophil extracellular traps (NETs) to eliminate foreign pathogens at the site of injury (**Figure 8**) (54). NETs aid by inhibiting inflammation along with neutrophil gelatinase-associated lipocalin (49).

NETs secretion and neutrophil degranulation are impaired in DFU; thus, leading to delayed wound healing (49, 54). Hyperglycemia upregulates the expression of neutrophil protein arginine deiminase (PAD)-4, inhibiting neutrophils from secreting NETs and consequently causing delayed wound healing (50). Diabetic wounds experience disruptions in phagocytosis, neutrophil degranulation, and the anti-infective effects of ROSs (49). *In vitro* studies have shown that the AGE-RAGE pathway, participating in impaired wound healing, compromised neutrophil regression, and showed early deficiency and regression of post-injury inflammatory factor release (53).

#### 4.2.3 Late inflammation phase: monocytes/macrophages

Monocytes and macrophages predominate in late inflammation phase of wound healing and macrophages are divided into two subtypes, M1 and M2. M1 subtype contributes by releasing inducible nitric oxide synthase which contributes to ROS formation, cytokines such as IL-1b, IL-12, IL-6, MMP-9, MMP-2, and TNF-α which promote a proinflammatory state (49, 55, 61). While M2 subtype promotes proliferation of cells and establishes an anti-inflammatory environment by releasing VEGF, TGF-β, IL-10 (49, 55), insulin-like growth factor 1 (IGF-1) and PDGF (61) as demonstrated in **Figure 8**.

**Figure 9:**
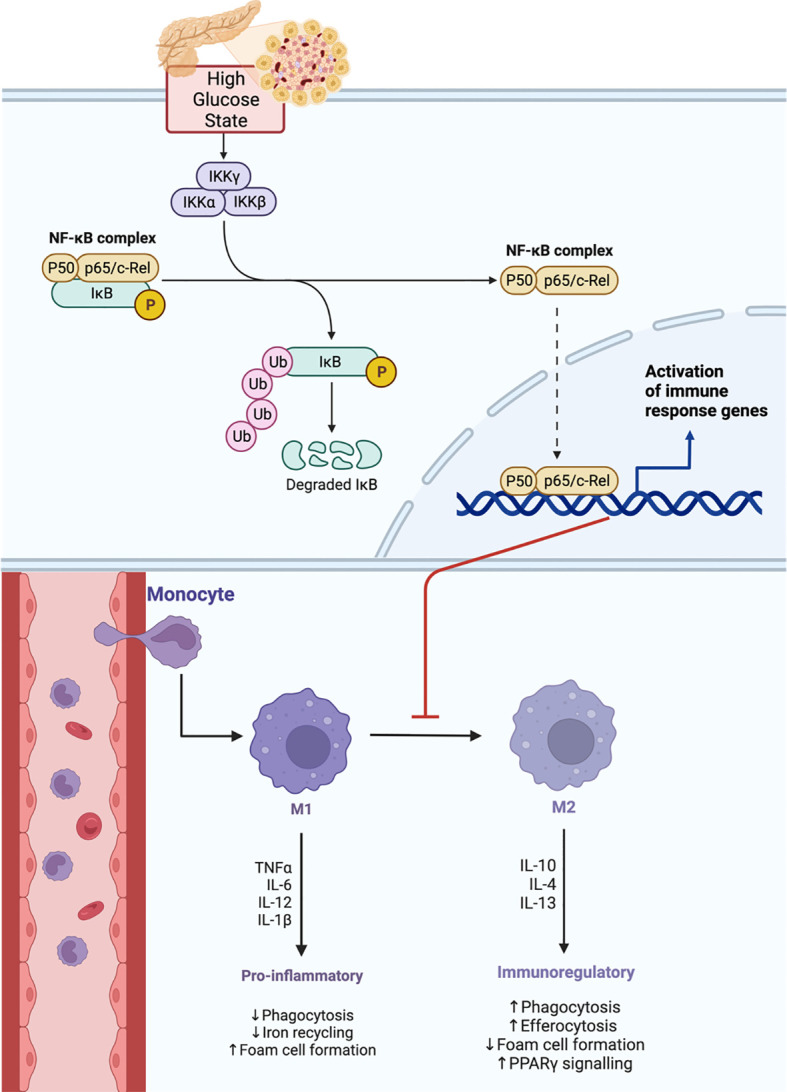
High glucose state effect on NF-kB signaling pathway disrupting macrophage phenotype transition.

It was shown that in hyperglycemic states, the macrophage phenotype is affected through the NF-kB signaling (**Figure 9**) (53). During DFU wound healing, macrophages subtype switch is impaired (49, 53, 55) yielding higher M1:M2 ratio (55, 61); thus, leading to a persistent proinflammatory state (49, 53, 55, 61). Moreover, AGEs inhibit wound healing by shifting the macrophage phenotypes to M1 (53). Additionally, macrophages physiological functions including ROS generation (49) and cytokine release (57) are disrupted during DFU healing. Furthermore, the phagocytic ability of macrophages in DFU wound is significantly reduced, resulting in ineffective removal of necrotic tissue.

#### 4.2.4 Late inflammation phase: lymphocytes

Following that, lymphocytes get involved in the late inflammatory phase of wound healing (**Figure 8**). T regulatory lymphocytes (Tregs) promote repair and regeneration by sustaining an anti-inflammatory state through releasing mainly TGF-β and IL-10 (61). Tregs maintain the macrophage phenotype transition through the suppression of IFN-γ release by other CD4+ effector T cells (61). Natural killer (NK) cells are also considered among T lymphocytes (62) as they contribute to the normal wound healing process via the release of certain proinflammatory cytokines, such as IFN-γ, or acting directly on target cells (62).

In DFU, there is an imbalance in the number of T lymphocytes favoring accumulation of effector T cells which promote inflammation, further complicating diabetic wound healing process (53). Although there is a decrease in NK cells number, IFN-γ is upregulated in diabetic wounds hence sustaining a proinflammatory state (56, 61). Furthermore, T-cell function abnormalities can result in decreased immune surveillance and poor immunological responses to infections. Therefore, this can aggravate persistent inflammation and impede wound healing.

#### 4.2.5 Proliferative phase: fibroblasts, keratinocytes, epithelial and endothelial cells

Subsequently, macrophage M2 subtype induce the proliferative phase by the release of growth factors, that activates fibroblasts, which play a role in the proliferation and production of ECM components in addition to assisting in the wound healing process (55). Fibroblasts mainly lay down types I and III collagen fibers at the site of injury, along with inflammatory cells, newly formed blood vessels, and endothelial cells resulting in granulation tissue formation (49, 55, 58). Later on, fibroblasts differentiate into myofibroblasts which are responsible for wound contraction (49, 55), secretion of proteases, MMPs, collagen and other ECM products (49) as shown in **Figure 8**. Type III collagen is replaced by type I collagen to increase tensile tissue strength (49). Moreover, keratinocytes migrate to injury site and differentiate to aid in reepithelization and covering of the granulation tissue (49, 55). Simultaneously, growth factors, such as EGF, FGF-2, and FGF-7 (KGF), are released from the injured epidermis to stimulate epithelial cell proliferation (55). Then epithelial cells migrate to injury site to cover the injured epidermis (58). Finally, endothelial cells proliferate to perform angiogenesis (58) which sustains fibroblast proliferation (55). EPC are recruited from the bone marrow to proliferate leading to *de novo synthesis* of blood vessels (55).

In DFU wound healing, there is impaired fibroblast proliferation and function. This could be due to the hyperglycemic state and accumulation of AGEs, which result in decreased fibroblast proliferation, increased fibroblast apoptosis, and inhibition of fibroblast migration to tissue (49). Fibroblasts of chronic wounds also show decreased expression of TGF-β receptors resulting in impaired downstream signaling (57). Furthermore, high glucose environment impairs the function of keratinocytes, hence, delaying wound reepithelization (49). Finally, EPC recruitment is impaired, therefore, delaying wound healing and blood vessel formation (49).

#### 4.2.6 Remodeling phase: MMPs

Finally, during the remodeling phase, the granulation tissue formed by fibroblasts undergoes a tightly regulated process of collagen synthesis and degradation mainly involving MMPs (54, 55, 58).

The inordinate activation of these proteases in DFU creates a highly proteolytic environment involving degradation of important growth factors and ECM proteins, in addition to impaired cell migration yielding a proinflammatory state with leukocyte infiltration leading to tissue damage and delayed wound healing (49, 57). The ECM becomes defective providing inadequate support to the wound at the site of the ulcer (54). To elaborate, excess presence of MMP-9 over a prolonged duration impedes the wound healing process by breaking down pivotal ECM proteins, such as fibronectin, that are crucial for effective wound recovery (**Figure 8**).

The healing of DFU does not progress through the normal physiological healing process, consequently leading to a vicious cycle of pathogenicity at different stages of wound healing (57, 61). During the inflammatory phase, contributors to this pathogenicity include neutrophilic abundance and dysfunction, alteration of macrophage phenotype transition, and increased secretion of proinflammatory cytokines. While in the proliferative phase, there is impaired fibroblast proliferation and migration, reduced growth factors and important ECM proteins, endothelial cells dysfunction, impaired keratinocytes differentiation and migration, and dysregulated angiogenesis at the site of wound injury. MMPs function is also compromised in the remodeling phase leading to the disorganized ECM granulation tissue formation (55).

## 5 Future perspectives

It is essential to understand the underlying pathophysiology of DFU in order to facilitate new therapeutic approaches to manage this condition. Current therapeutic approaches include wound debridement, negative pressure wound therapy, and antimicrobial therapy. It is crucial to comprehend between different factors that contribute to DFU and its delayed wound healing which include oxidative stress, diabetic neuropathy, dysregulated angiogenesis, and impaired immune response. We propose to direct the management by targeting these aforementioned components to aid in better wound healing for the sake of enhancing patient quality of care and reducing complications. Targeting PKC, AGE-RAGE, and the polyol pathway, as well as the intracellular pathways involved in the pathogenesis of DFU such as the NF-kB and MAPK would be the optimal approach to prevent and treat DFU. Moreover, immunological therapy can be implemented to rectify the impaired immune response.
